# Identifying Maternal Constraints on Fetal Growth and Subsequent Perinatal Outcomes Using a Multiple Embryo Implantation Model

**DOI:** 10.1371/journal.pone.0166222

**Published:** 2016-11-08

**Authors:** Nigel Pereira, Tyler Cozzubbo, Stephanie Cheung, Zev Rosenwaks, Gianpiero D. Palermo, Queenie V. Neri

**Affiliations:** Ronald O. Perelman and Claudia Cohen Center for Reproductive Medicine, Weill Cornell Medical College, New York, NY, United States of America; INIA, SPAIN

## Abstract

**Introduction:**

Although the majority of singleton births after in vitro fertilization (IVF) are uncomplicated, studies have suggested that IVF pregnancies may be independently associated with low birth weight (LBW), preterm birth (PTB), and perinatal mortality. These outcomes complicate multiple gestations as expected, but have also been reported in singletons. A multiple embryo implantation model allows for assessment of the early in utero environment, and therefore, assessment of any maternal constraints on developing fetuses. We question whether adverse perinatal outcomes associated with assisted reproductive techniques (ART) occur as a result of maternal physiologic adaptations.

**Patients and Methods:**

This is a retrospective, single center study of ART cycles, specifically intracytoplasmic sperm injection (ICSI) cycles during a 16-year period. For each positive pregnancy test 9–11 days after embryo transfer, an ultrasonogram was performed at 7 weeks of gestation to record the number of implanted fetal poles with cardiac activity. Controlled ovarian stimulation (COS), hCG trigger, oocyte retrieval and sperm injection were performed as per our standard protocols. First trimester implantation sites that resulted in live births were defined as “true” to distinguish them from those that spontaneously reduced called “virtual.” Birth outcomes analyzed included birth weight and gestational age at delivery.

**Results:**

A total of 17,415 cycles were analyzed. The average maternal age was 36.9 (±5.0) years. An overall fertilization rate of 73.4% generated approximately 48,708 good quality cleavage-stage embryos. In most patients (92.8%), an average of 3 embryos were transferred. The clinical pregnancy rate was 39.2% (n = 6,281). The overall occurrence of multiple gestations was 38.2% (n = 2,608) consisting of 2,038 twin, 511 triplet, and 59 quadruplet pregnancies. Of these multiple gestations, 18.6% of twin, 54.2% of triplet and 76.3% of quadruplet gestations spontaneously reduced. Failure of the implanted embryo to progress was not related to maternal age. Singleton newborns resulting from multiple implantation sites had lower birth weights (*P*<0.01) and shorter gestational ages (*P*<0.01) than those from a single implanted embryo. The number of embryos transferred did not affect the gestational length of singleton newborns. Although the birth weights of singletons from multiple implantation sites (virtual singletons) were lower than true singletons, the birth weight of virtual singletons were comparable to the birth weights of true twin, triplet, and quadruplet live births. Multiple logistic regression revealed that virtual singletons were an independent risk factor for PTB (odds ratio: 4.55, 95% CI 2.23–9.29) and LBW (odds ratio: 3.61, 95% CI 1.78–7.32), even after controlling for the number of oocytes, stimulation protocol type, sperm source, total gonadotropins administered, age, embryo quality, and day of embryo transfer.

**Conclusions:**

Our study highlights that embryonic implantation sites during early gestation set the growth profile of each embryo, dictating later growth patterns. Specifically, spontaneous reduction of an embryo after multiple embryo implantations can confer greater perinatal risk in the form of LBW and PTB to the surviving fetus. Our findings suggest that maternal constraints or physiologic adaptations maybe one of the mechanisms mediating adverse perinatal outcomes when multiple embryo implantation occurs.

## Introduction

The use of assisted reproductive techniques (ART) to overcome infertility has gained popularity over the past 2 decades. A total of 160,521 ART procedures were performed in 2013 across 467 fertility clinics in the United States, resulting in 1.6% of all live births [1]. Based on global statistics reported in 2015, more than 1,251,881 ART procedures were performed that resulted in 229,442 live born infants [2]. With the increasing utilization of ART, there has been a corresponding emergence of evidence linking ART to adverse perinatal outcomes such as preterm birth (PTB), low birth weight (LBW), very low birth weight (VLBW), congenital malformations, and developmental delays [3–5]. While multiple gestations have been habitually implicated in the pathogenesis of these adverse outcomes, recent studies have highlighted that such outcomes maybe recurrent in singleton gestations as well [6–8].

Intriguingly, previous studies have reported that singletons resulting from single embryo transfer (SET) have a higher birth weight than those resulting from double-embryo transfer [9,10]. Furthermore, singletons resulting from twin embryo implantation have a two-fold increase in the occurrence of small for gestational age (SGA) when compared to “true singletons” i.e., those resulting from a single implanted embryo [11]. The rate of LBW among ART singletons also positively correlates with the increasing number of fetal heartbeats detected at first trimester ultrasonography [12,13]. These findings suggest that growth profile of each implanted embryo is perhaps dictated by the in utero environment very early in gestation. Thus, the primary objective of the current study is to investigate whether early in utero maternal physiologic adaptations mediate the development of adverse perinatal outcomes in ART pregnancies. For the purpose of the study, we utilize a multiple embryo implantation model, which allows for assessment of the early in utero environment, and therefore, assessment of any maternal constraints on the growth or gestational age profiles of the developing embryo.

## Patients and Methods

### Cycle Inclusion Criteria

The Weill Cornell Medicine institutional review board approved our retrospective study protocol (IRB#1503016064). Patients initiating intracytoplasmic sperm injection (ICSI) cycles at the Ronald O. Perelman and Claudia Cohen Center for Reproductive Medicine resulting in fresh embryo transfer (ET) during a 16-year time-period were analyzed for potential inclusion. All patients undergoing frozen-thawed ET, utilizing surgically-retrieved sperm, or those utilizing donor oocytes were excluded. Patients with a positive pregnancy test on cycle day (CD) 28 i.e., 14 days after oocyte retrieval underwent transvaginal ultrasonography (TVUS) on CD49 to record the implanted embryos with cardiac activity.

To address our question, we grouped ICSI cycles according to the initial number of embryos transferred, those that actually implanted, and the actual number of live birth(s). ICSI cycles with the number of implanted embryos with cardiac activity on CD49 consistent with the number of live births were defined as “true” live births to distinguish them from “virtual” live births which were associated with spontaneously reduced embryos during the first trimester. In other words, a singleton live birth was considered a true singleton when only one implanted embryo with cardiac activity was noted on CD49. In contrast, a singleton live birth after visualization of >1 embryo with cardiac activity was considered a virtual singleton. For the purpose of the study, only those implanted embryos that spontaneously reduced within the first trimester were included. Although all patients with selective reduction were excluded from the final analysis, a separate sub-analysis was carried out to compare the birth weights and gestational age at delivery for these patients.

### Ovarian Stimulation, Sperm Injection and Laboratory Protocols

Controlled ovarian stimulation (COS) and oocyte retrieval were performed per our standard protocols [14]. Gonadotropin dosing was based on patient age, weight, antral follicle count, serum anti-müllerian hormone (AMH) level, and previous response to stimulation. hCG was used as the ovulatory trigger and was administered according to a sliding scale [14]. The hCG trigger was administered when the two lead follicles attained a mean diameter >17 mm. Oocyte retrieval was performed under transvaginal ultrasound guidance with conscious sedation approximately 34–35 hours after hCG administration. Daily intramuscular progesterone (50 mg) was begun the day after oocyte retrieval. Oocytes were exposed to 40 IU recombinant hyaluronidase (Cumulase, Halozyme Therapeutics Inc., San Diego, CA, USA) to remove the cumulus-corona complex [15,16].

Semen samples produced after 2–5 days of abstinence were evaluated for volume, total count, concentration and motility using WHO criteria [17]. Sperm microinjection was carried out based on previously described protocols [18]. Oocytes were examined 12–17 hours after ICSI for normal fertilization and the resulting embryos were incubated in in-house culture media. Patients underwent embryo transfer on day 3 or day 5. Day 3 embryos were graded based on the Veeck criteria [19], while the blastocyst-stage embryos were graded based on criteria described by Gardner and Schoolcraft [20]. Embryo transfers were performed with Wallace catheters (Smiths Medical Inc., Norwell, MA, USA) at approximately 1 cm less than the uterine depth identified at prior trial transfer.

### Outcome Variables

Baseline demographics of age and body mass index (kg/m^2^) were recorded for the study cohort. In addition, ICSI characteristics recorded were total gonadotropins administered (IU), oocytes retrieved per ICSI cycle, oocytes injected per ICSI cycle, fertilization rate, mean number of embryos transferred, and day of ET. Fertilization rate was defined as the number of 2 pronuclear (2PN) embryos out of the total number of mature oocytes injected. Implantation rate was defined as number of intrauterine gestations with fetal cardiac activity detected by TVUS on CD49 out of the total number of embryos transferred. Clinical pregnancy rate was defined as the number of intrauterine gestations with fetal cardiac activity on CD49 per ICSI cycle. Any loss of pregnancy after the visualization of an intrauterine gestation with cardiac activity was considered a spontaneous miscarriage. Any birth after 24 weeks of gestational age was considered a live birth. Live birth characteristics analyzed were overall birth weight, low birth weight (LBW), very low birth weight (VLBW), incidence of term birth, and incidence preterm birth (PTB). Birth weight <2,500 g and <1,500 g irrespective of gestational age was considered LBW and VLBW [21]. Any live birth >37 weeks of gestational age was considered term birth, while live birth ≤37 weeks of gestational age was defined as PTB [22].

To assess the development of ART offspring in the study cohort, consenting parents of all children aged 3 years (± 6 months) were asked to complete Ages & Stages Questionnaires (ASQ^®^), which are geared towards learning about their cognitive abilities, socio-emotional development, and motor skills (IRB#0705009149). The ASQ^®^, is a series of parent-completed developmental questionnaires spanning from birth to 5 years of age [23] to screen five key developmental areas—communication, gross motor, fine motor, problem solving, and personal-social—and has an overall section addressing specific parental concerns. According to the child's score, questionnaires were ranked as showing a typical development or as needing further evaluation (i.e. 'at risk', clinical range) or intervention [24]. The ASQ^®^ has been standardized for the general population and corrected for ART children [25].

### Statistical analysis

Non-parametric variables were expressed as median (interquartile range [IQR]), while categorical variables were expressed as number (n) and percentage of occurrence (%). Continuous variables were expressed as mean ± standard deviation (SD). Wilcoxon rank-sum tests, McNemar’s chi-square (χ2) tests and Independent *t*-tests were utilized for non-parametric, categorical, and continuous variables, respectively. Multiple logistic regression was used to explore the association of LBW and PTB with the following variables: true singleton vs. virtual singleton; number of oocytes (<10 vs. >10); stimulation protocol (gonadotropin releasing hormone-agonist vs. gonadotropin releasing hormone-antagonist based); sperm source (ejaculated vs. surgically retrieved); total gonadotropins administered (<2500 IU vs. 2500–4000 vs. >4000); age (<35 years vs. 35–37 vs. 30–40 years); embryo quality (grade 1 vs. 1.5 vs. 2); and day of embryo transfer (day 3 vs. day 5). All statistical analyses were performed using STATA version 13 (College Station, TX: StataCorp LP), with statistical significance set at *P*<0.05.

## Results

A total of 17,415 ICSI cycles were evaluated (Table 1).

**Table 1:** Overall ICSI cycle outcomes of the study cohort.

No. of (%)	ICSI
Cycles	17,415
Maternal age (years)	36.9 ± 5
Body mass index (kg/m^2^)	22.9 ± 6.55
Gonadotropins administered (IU)	2443.5 ± 1853.2
Oocytes retrieved per ICSI cycle	10.3 ± 6.49
Oocytes injected per ICSI cycle	9.99 ± 6.15
Fertilization	106,720/145,368 (73.4%)
Embryos transferred	48,708
Day 3	15,836 cycles (90.9%)
Day 5	1,579 cycles (9.1%)
Mean embryos transferred	3.01
Clinical pregnancy	6,821 (39.2%)
Spontaneous miscarriage	891 (5.12%)
Biochemical pregnancy	2,019 (11.6%)
Live birth	5,725
Live born	7,817

Data are presented as mean ± (standard deviation), n (%) and median (interquartile range).

In these cycles, where the average age of the women was 36.9 ± 5 years (range 18–49), an overall fertilization rate of 73.4% generated 48,708 good quality embryos. Of all ICSI cycles, 15,836 (90.9%) cycles resulted in day 3 ET, while the remaining 1,579 (9.1%) cycles resulted in day 5 ET. An average of 3.01 embryos were transferred per ICSI cycle. The clinical pregnancy and spontaneous miscarriage rates for the study cohort were 39.2% and 5.12%, respectively. There were 5,725 live births, which resulted in birth of 7,817 live born neonates. The overall occurrence of multiple gestations was 38.2% (n = 2,608) consisting of 2,038 twin, 511 triplet, and 59 quadruplet pregnancies. Of these multiple gestations, 18.6% of twin, 54.2% of triplet and 76.3% of quadruplet gestations spontaneously reduced (*P*<0.01). The rate of spontaneous miscarriage in all singleton gestations was 7.9%, while the spontaneous miscarriage in twin, triplet and quadruplet gestations with spontaneous embryo reduction was 7.4%, 9.9%, and 14%, respectively. In spite of a comparable maternal age, the proportion of pregnancies presenting with spontaneous in utero reduction correlated with the number of embryos implanted (Table 2). The failure of the implanted embryo to progress was not related to maternal age (*P* = 0.14) or gonadotropins administered (*P =* 0.60).

**Table 2:** Incidence of spontaneous in utero reduction of an implanted embryo according to the number of embryos implanted.

Parameter	Number of fetal heartbeats				
	1	2	3	4	*P*
	(n = 4,213)	(n = 2,038)	(n = 511)	(n = 59)	
Maternal age (years)	36.1 ± 5	36.1 ± 4	35.6 ± 4	36.0 ± 3	0.14
Gonadotropins administered	2358.1	2406.8	2427.9	2324.2	0.52
Oocytes injected	10.1 ± 6	10.3 ± 6	10.7 ± 6	10.2 ± 6	0.18
Embryos transferred	12,820	6,288	1,793	251	—
Mean embryos transferred	3.0	3.1	3.5	4.2	<0.01
In utero reduction	—	379 (18.6%)	277 (54.2%)	45 (76.3%)	<0.01

Data are presented as mean ± (standard deviation), n (%) and median (interquartile range).

**Fig 1:**
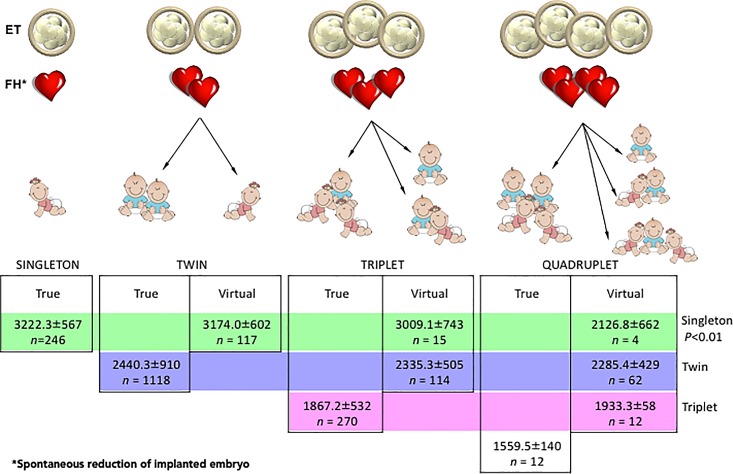
Birth weight of offspring ranked according to the increasing number of embryos transferred (ET) and implanted as characterized by a fetal heartbeat (FH). Birth weight of singletons, twins and triplets derived from an equal number of embryo implanted at first trimester ultrasound were compared in a color-coded fashion across with those of pregnancies with vanishing co-twins.

**Fig 2:**
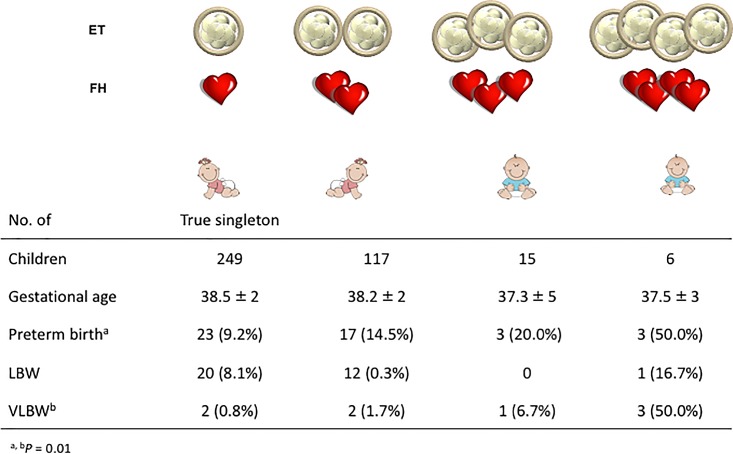
Singletons were ranked according to the increasing number of embryos transferred and implanted at first trimester ultrasonography. Across the implantation order, gestational age, the incidence of preterm birth, low birth weight (LBW), and very low birth weight (VLBW) were compared. ET = embryos replaced; FH = fetal heartbeat.

**Fig 3:**
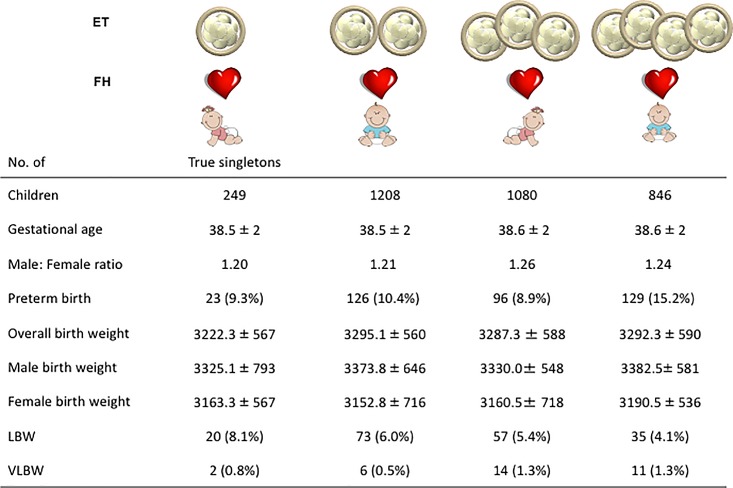
Singletons were ranked according to the increasing number of embryos transferred and that yielded only a single implantation at first trimester ultrasound. For each embryo transfer cohort, gestational age and incidence of preterm birth were compared. Similarly, across the increasing number of embryos transferred, we compared the birth weight together with the proportion of low birth weight (LBW) and very low birth weight (VLBW). Sex ratios were also compared. ET = embryos replaced; FH = fetal heartbeat.

To investigate our hypothesis on the constraints exerted by the supernumerary embryos implanted, all embryos that were transferred and successfully implanted were ranked according to the increasing gestational order (Fig 1). The birth weight of live borns was recorded in relation to the number of implanted embryos at the CD49 TVUS. As evident in Fig 1, singleton newborns resulting from multiple implantation sites i.e., virtual singletons had lower birth weights (*P*<0.01) than those from a single implanted embryo i.e., true singleton. This, however, was not true when true twins were compared to virtual twins, or when true triplets were compared to virtual triplets (Fig 1). When the length of gestation of singleton deliveries that originated from increasing number of implanted embryos was compared, there was no clear correlation with gestational age (Fig 2). However, the PTB rate across those implantation orders increased progressively with increasing number of embryos implanted (*P*<0.01). A similar trend was observed for the rates of LBW and VLBW infants with increasing implantation order (*P*<0.01, Fig 2), indicating that virtual singletons yielded a high proportion of small for gestational age infants. In contrast, singletons resulting from >1 embryo transferred, but from a single implantation site showed no difference in the gestational age at delivery, overall birth weight, PTB rate, LBW rate, or VLBW rate (Fig 3). Furthermore, the male: female sex ratio, male birth weight and female birth weight of all singletons were comparable, irrespective of the number of embryos transferred. Based multiple logistic regression analysis, it is also evident that virtual singletons are an independent risk factor for PTB (odds ratio: 4.55, 95% CI 2.23–9.29) and LBW (odds ratio: 3.61, 95% CI 1.78–7.32), even after controlling for the number of oocytes, stimulation protocol type, sperm source, total gonadotropins administered age, embryo quality, and day of embryo transfer (S1 Fig and S2 Fig).

**Fig 4:**
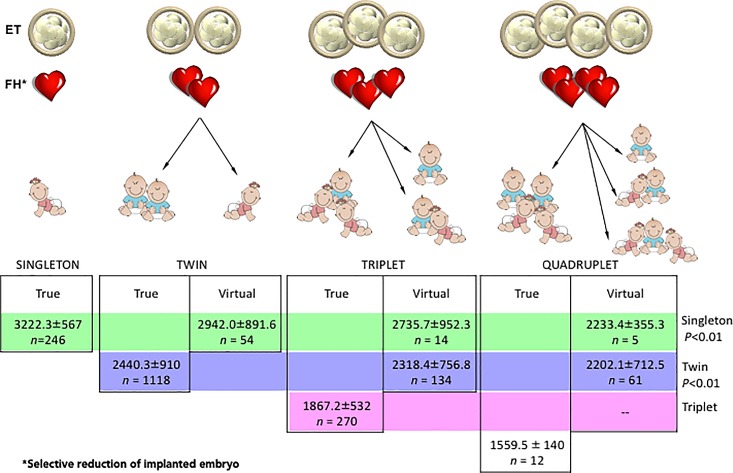
Birth weight of offspring ranked according to the increasing number of embryos transferred (ET) and implanted as characterized by a fetal heartbeat (FH). Birth weight of singletons and twins derived from selective reduction of higher order implantations were compared.

The birth outcomes and gestational age at delivery for patients undergoing selective fetal reduction was also assessed (Fig 4). A total of 261 patients underwent selective reduction– 54 from a twin to singleton gestation, 14 from a triplet to singleton, 134 from a triplet to twin gestation, 5 from a quadruplet to singleton gestation, and 61 from a quadruplet to twin gestation. The birth weights of singletons and twins decreased with increasing gestational order at selective reduction (*P*<0.001). The mean gestational age at delivery for the singleton group was 38.5 weeks and for the twin group was 35.9 weeks after selective reduction.

To assess the effect of high order embryo implantations on long-term developmental outcomes, 3-year-old children were assessed for psychological and motor development through a parent-administered ASQ^®^ survey (Table 3).

**Table 3:** Cognitive function according to gestational order of true versus virtual gestations.

	Singletons		Twins		Triplets	
No. of (%)	True	Virtual	True	Virtual	True	Virtual
At risk	24 (8.9)	5 (12.8)	15 (8.4)^a^	8 (30.8)^b^	11 (18.3)	0
Normal	245	34	164	18	49	3
total	269	39	179	26	60	3

^a vs b^χ^2^, 2x2, 1*df*.

Five-hundred-eighty-nine children were recruited, of which 13 were born after selective reduction, and were therefore excluded. With the remaining 576 children, the large majority (n = 508) fell into the category of true singletons, twins and triplets, while the remaining 68 children belonged to the category of virtual gestations. The cognitive functions of true singletons and true twins were within the normal limits. In contrast, those derived from multiple embryo implantation sites had a higher incidence of being at risk for cognitive delays (*P* = 0.0007, Table 3).

## Discussion

Promoting the health of ART offspring continues to be a central facet of the practice of reproductive medicine. Different factors such as the underlying cause of infertility, advanced maternal age, ART technique, the non-physiological environment where gametes or embryos are nurtured, as well as multiple gestations have been implicated in the pathogenesis of adverse perinatal outcomes in ART pregnancies. Most unsettling, however, are emerging data suggesting that even singleton ART pregnancies maybe at risk for PTB, LBW or VLBW compared to naturally conceived pregnancies.

A common criticism within the ART is the practice of transferring a higher number of embryos, suggesting that the aforementioned perinatal risks may be generated iatrogenically [26]. Evidence for this stems from monotocous species where fetal growth can be influenced or possibly limited by maternal body size [27]. This mechanism is often called ‘maternal constraint’ [27] and was first described in cross-breeding studies of horses and ponies [28]. Based on the observation that a fetus would grow bigger in the uterus of large-breed animal than that of a smaller animal, it was hypothesized that fetal growth was constrained by maternal phenotype, and specifically by uteroplacental factors [29]. Thus, maternally-driven fetal growth patterns prevent fetal overgrowth and consequent dystocia, thereby promoting species survival, which would be otherwise compromised if the fetus outgrew the mother's reproductive tract [27,28]. Interestingly, maternal height seems to be a major determinant of fetal size [30]. Fetal size, to some extent, may also be constrained by factors such as maternal age and parity [28]. In pregnancies involving ovum donation the only contributing factors to newborn birth weight were gestational age and the recipient mother’s weight, whereas the weight of the donor mother had no role [31].

Plasticity in placental endocrine and metabolic function is likely to play an important role in the ability of the fetus to accommodate itself in the intrauterine environment while still satisfying the demand-driven fetal constraints. For example, twin fetuses grow in the same uterus in a space usually occupied by one fetus. Yet, there are instances where differences in implantation sites, in utero locations, as well as subsequent placentation may result in greatly differing birth weights, despite similar gestational age [32]. Thus, it is important to analyze the factors limiting the growth of multiple implanted embryos. Implantation is a highly regulated process coordinated between the embryo and the uterus during early gestation, ultimately leading to invasion of the blastocyst in the endometrium, placentation and eventual fetal growth [33–35]. In polytocous species, embryos are generally distributed evenly along the uterus rather than at random implantation sites [35]. This intriguing phenomenon of equidistant embryo distribution along the uterine horn, in the presence of multiple implantation sites, is generally limited to rodents with multiple implantation sites [36]. However, one may posit that the mechanisms underlying embryo spacing in rodents may contribute to proper embryo localization and positioning in single-birth species such as humans [36]. In human beings, several mechanical, cellular, and molecular factors have been implicated in restricting embryo implantation to the uterine fundus [36,37]. High resolution ultrasonographic studies have suggested well-regulated wave-like movements from the cervix to fundus within the pre-implantation uterine environment [37]. These well-regulated waves likely promote proper localization of the embryo within the uterus, while aberrant waves could result in sub-optimal implantation, thereby increasing the risk of miscarriage, or implantation at non-physiologic sites such as the fallopian tube leading to ectopic pregnancy [36,37]. Disruption of cellular factors such as the β2-adrenoreceptor (β2-AR) [36], or lysophosphatidic acid (LPA) and its receptor LPA3 [35] have shown to disrupt proper embryo spacing and implantation in rodent models, culminating in either early or mid-gestation pregnancy loss. Recent data also suggests that proper embryo localization and perhaps implantation occurs through embryonic-maternal cross-talk via exosomes, which are cell-derived vesicles of 30–100 nm in diameter containing proteins, messenger RNA (mRNA), microRNA, and DNA, and have pleiotropic effects on both embryonic and maternal environments [38].

Several investigators have theorized as to how physiologic adaptations may occur in utero upon spontaneous reduction of an implanted embryo. For example, La Sala et al. [39] suggest that a spontaneously reduced embryo may cause shunting of blood from the placenta of the surviving twin via vascular anastomoses. Another proposition is that chronic inflammation of the surviving twin’s placenta ensues after the reduction of an embryo [40]. However, one may also postulate that the mechanisms regulating embryo growth once overcrowding of the uterus has been detected is likely triggered at the time of implantation and not earlier. In fact, the establishment of embryo spacing would require the transferred embryos to communicate with the endometrium or with each other to achieve a putative domain. It is interesting to note that the weights of singletons resulting from a single implantation was not affected by the number of embryos that failed to implant, even after controlling for maternal age, suggesting that those embryos did not alter the endometrium.

Many phenotypic changes in early life are the result of developmental plasticity and its appearance may follow an initiating event, sometimes after a substantial delay. However, at times phenotypic changes induced in utero or in the neonate may confer disadvantages. There is much evidence that a suboptimal fetal environment, as reflected in smaller size at birth, is linked to increased risk of coronary heart disease and type 2 diabetes [41,42]. Diverse effects on the fetus can have physiological consequences not reflected in birth size, and in early gestation, environmental cues can influence development without affecting birth weight. Such developmental origins of disease have been termed 'fetal programming' [41], because they suggest that events occurring early in life may alter subsequent development. Furthermore, factors acting in the peri-conceptional period affect genomic imprinting [43] and other epigenetic processes, hormone receptor development and embryo/trophoblast cell allocation [44], whereas cues later in fetal development alter structural and or functional differentiation of tissues. This is further confirmed in our study suggesting that the early stress represented by the multiple implants yielded a surviving cohort affected by an increased risk for developmental delays as seen in twins derived from higher order implants.

The noticeable strengths of our study include the 16-year-long study period, large sample size of 17,415 ICSI cycles utilizing standardized ovarian stimulation protocols, as well as the use of the ASQ^®^ to report the developmental outcomes of children conceived via ICSI. Furthermore, our logistic regression analysis confirms the risks of PTB and LBW in singleton newborns resulting from multiple implantation sites than those from a single implanted embryo. However, our study is not without limitations. First, only ICSI cycles were included in the current study, which reflects up to 80% of the overall patient population undergoing ART at our center. However, it is worthwhile to note that similar trends in PTB and LBW have been reported in patients undergoing in vitro fertilization using smaller sample sizes in internal [45] and external investigations [39,40]. Second, a mean of 3.01 embryos were transferred in the current study. Second, a mean of 3.01 embryos were transferred in the current study. While this number purely reflects the long study duration of 16 years, it is encouraging to note that the mean number of embryos transferred per ICSI cycle has decreased from 3.35 to 2.27 during the study period (S3 Fig). Third, only embryos that spontaneously reduced within the first trimester were included in the study. It is possible that spontaneous reduction of embryos later in the first trimester or early in the second trimester may result in physiologic adaptations dictating greater fetal growth abnormalities in the surviving twin. Fourth, the ASQ^®^, which can be biased due to parental reporting, was used to track the developmental outcomes of the children conceived via ICSI. Due to the fact that our center is a specialized referral center, a majority of our patients return to their home state or to their country of residence following ICSI treatment. Thus, administration of the Bayley Scales of Infant Development (BSID) or the Bayley Mental Development Index (MDI), in person, is limited by logistical or travel reasons. In one cross-sectional study, 124 and 112 infants were assessed at 12 and 24 months after birth with the ASQ^®^ and BSID [46]. At 12 months, the ASQ^®^, had a low sensitivity (0.20–0.60) in identifying infants with mental delay. Although the specificity (0.90–0.97) was better for the psychomotor scale, the sensitivity remained insufficient (0.25–0.52). At 24 months, the ASQ^®^, had good sensitivity (0.75–0.92) and specificity (0.55–0.78) for detecting mental delays. However, the sensitivity (0.31–0.50) remained unsatisfactory for detecting motor delays. At least 2 other studies [47,48] have confirmed that only a moderate correlation exists between ASQ^®^ and BSID because of which the ASQ^®^ cannot substitute for the BSID. Despite these shortcomings, it is important to note that the ASQ^®^ has been standardized for the general population and corrected for ICSI children up to the age of 5 years [25] and our center has previously used this tool to report the developmental outcomes of 5,891 neonates conceived from 12,866 consecutive ICSI cycles [24]. Finally, it is important to note that maternal constraints maybe one of the many mechanisms mediating adverse perinatal outcomes when multiple embryo implantation occurs in humans. Furthermore, multiple gestations are independently associated with adverse perinatal outcomes such as LBW and PTB, which thereby increase the risks of adverse developmental or cognitive outcomes. Thus, caution must be exercised when interpreting the perinatal and developmental outcomes of true or virtual twin, triplet or quadruplet gestations.

## Conclusions

In conclusion, our study highlights that embryonic implantation sites during early gestation set the growth profile of each embryo, dictating later growth patterns. Specifically, singleton newborns resulting from multiple implantation sites had lower birth weights and shorter gestational ages than those from a single implanted embryo. Furthermore, the rates of PTB, LBW, and VLBW progressively increased with increasing implantation order. Finally, singleton newborns from multiple implantation sites had higher independent risks of PTB and LBW, even after accounting for different variables with multiple logistic regression analysis.

## Supporting Information

S1 Fig Results of multiple logistic regression exploring the association of preterm birth (PTB) with the following variables: true singleton vs. virtual singleton; number of oocytes; stimulation protocol; sperm source; total gonadotropins administered; age; embryo quality; and day of embryo transfer. (TIFF)

S2 Fig Results of multiple logistic regression exploring the association of low birth weight (LBW) with the following variables: true singleton vs. virtual singleton; number of oocytes; stimulation protocol; sperm source; total gonadotropins administered; age; embryo quality; and day of embryo transfer. (TIFF)

S3 Fig Mean number of embryos transferred during the study duration. (TIFF)
