# Correction: Identifying Maternal Constraints on Fetal Growth and Subsequent Perinatal Outcomes Using a Multiple Embryo Implantation Model

**DOI:** 10.1371/journal.pone.0168327

**Published:** 2016-12-09

**Authors:** The PLOS ONE Staff

## Notice of Republication

This article was republished on November 18, 2016, to correct an error where an ORCID iD was erroneously assigned to Nigel Pereira, which was introduced during the typesetting process. The publisher apologizes for the error.
