# Supplementary figures and images for: Identifying Maternal Constraints on Fetal Growth and Subsequent Perinatal Outcomes Using a Multiple Embryo Implantation Model
Source: PLoS One. 2016 Nov 8;11(11):e0166222. doi: 10.1371/journal.pone.0166222 (PMC5100992; doi:10.1371/journal.pone.0166222)

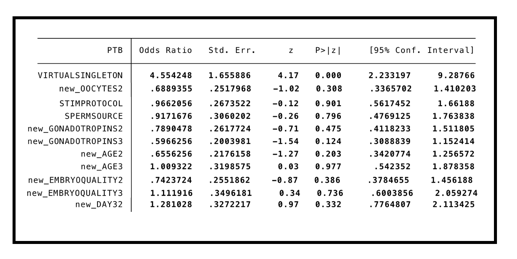

Supplement: S1 Fig — (TIFF) [file pone.0166222.s001.tiff]

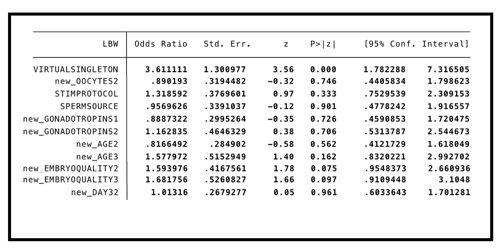

Supplement: S2 Fig — (TIFF) [file pone.0166222.s002.tiff]

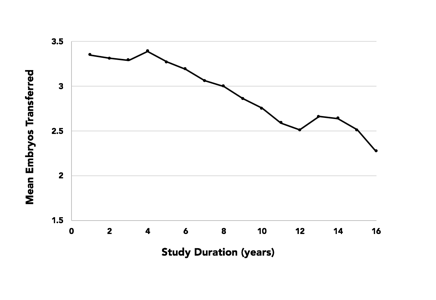

Supplement: S3 Fig — (TIFF) [file pone.0166222.s003.tiff]
